# Association analysis of mitochondrial genome polymorphisms with backfat thickness in pigs

**DOI:** 10.1080/10495398.2023.2272172

**Published:** 2023-11-15

**Authors:** Hao Liu, Xing Zhang, Yaning Hu, Xingbo Zhao

**Affiliations:** a State Key Laboratory of Animal Biotech Breeding, China Agricultural University, Beijing, China; b MOE Key Laboratory for Biosystems Homeostasis and Protection and Innovation Center for Cell Signaling Network, Life Sciences Institute, Zhejiang University, Hangzhou, Zhejiang, China; c Guangdong Provincial Key Laboratory of Animal Molecular Design and Precise Breeding; School of Life Science and Engineering, Foshan University, Foshan, China; d School of Biology and Biological Engineering, South China University of Technology, Guangzhou, China

**Keywords:** Backfat thickness, mitochondrial DNA, polymorphism, swine

## Abstract

Mitochondrial DNA (mtDNA) variations and associated effects on economic traits have been widely reported in farm animals, as these genetic polymorphisms can affect the efficiency of energy production and cell metabolism. In studies related to metabolism, the deposition of fat was highly correlated with mitochondria. However, the effect of mtDNA polymorphisms on porcine backfat thickness (BFT) remained unclear. In this study, 243 pigs were collected to analyse the relationship between BFT and mtDNA polymorphisms. There were considerable differences in BFT, ranging from 5 mm to 18 mm. MtDNA D-loop sequencing discovered 48 polymorphic sites. Association analysis revealed that 30 variations were associated with BFT (*P* < 0.05). The polymorphism m.794A > G showed the maximum difference in BFT between A and G carriers, which differed at ∼2.5 mm (*P* < 0.001). The 48 polymorphic sites generated 22 haplotypes (H1-H22), which clustered into 4 haplogroups (HG1-HG4). HG1 had a lower BFT value than other three haplogroups (*P* < 0.01), whereas H4 in HG1 exhibited the lowest BFT of all haplotypes analyzed (*P* < 0.01). The results of this study highlight an association between mtDNA polymorphisms and BFT, and suggest the potential application of mtDNA in pig molecular breeding practices.

## Introduction

Mitochondria are organelles found in most eukaryotic cells, including those of plants and animals, which are commonly referred to as powerhouses.^1^ Mitochondrial function is crucial for many biological processes, including calcium homoeostasis, hormone synthesis, regulation of reactive oxygen species, metabolism, cell signalling, autophagy and apoptosis,^2^ while dysfunctional mitochondria are implicated in diseases, such as diabetes.^3^ In addition, mitochondria are primary organelles for fatty acid oxidation and provide precursor molecules for lipid metabolism, suggesting that being associated with fat deposition.^4^^,^^5^ MtDNA is the genetic material found in mitochondria, which encodes proteins involved in mitochondrial respiratory chain complexes. Accordingly, mutations in mtDNA have been correlated with economic traits closely related to energy in farm animals, including bovine stress resistance,^6^ milk yield,^7^ beef marbling score and longissimus muscle area,^8^ chicken chest dept,^9^ ovine prolificacy,^10^^,^^11^ and porcine intramuscular fat,^12^ litter size,^13^ muscle depth, lifetime daily gain and teat quality.^14^

Adipose tissue is a specialized type of connective tissue that plays a crucial role in energy storage and metabolism in animals.^15^^,^^16^ In adipocytes, the process of lipogenesis refers to the synthesis or production of fatty acids and triglycerides, which plays a vital role in regulating metabolic homoeostasis, energy balance, and body composition.^17^^,^^18^ It mainly involves the conversion of excess glucose, dietary carbohydrates, and other substrates into fatty acids, which are then stored as triglycerides in adipose tissue. In pigs, BFT is a crucial indicator that reflects the content of adipose tissue present. BFT is a measure of the amount of fat located between the skin and muscle tissue on the back of an animal, typically pigs or cattle, which is an important quantitative trait and varies significantly among different breeds.^19–21^ It is an important indicator for grading beef quality^22^^,^^23^ and selecting pigs with high lean meat percentages. Besides, BFT is related to reproductive performance, lactation capacity, feed utilization rate, carcass grading, and meat quality.^24–26^ Evidence suggests that mtDNA polymorphisms can play a role in fat deposition and obesity in animals. Studies have shown that mtDNA mutations are associated with mitochondrial dysfunction, which leads to a decrease in energy production and an imbalance in energy intake and expenditure, resulting in obesity.^27–29^ One study has shown that Qatari individuals with mitochondrial haplogroup J have an increased risk of obesity, whereas individuals with haplogroup X own a lower risk for obesity.^30^ Overall, although the exact mechanisms by which mtDNA influences fat deposition are not fully understood, there is evidence to suggest that variations in mtDNA can play an important role in the regulation of fat accumulation in animals.

Currently, with the development of molecular biology technology, many candidate genes related to backfat thickness and their molecular markers have been identified.^31^^,^^32^ Yet, as the important genetic material inherited through the maternal line, mitochondrial genome contributions to the dynamics of live BFT in pigs remain unknown. Thus, the present study explored the relationship between mtDNA polymorphisms and live BFT in pigs, aimed to provide useful markers for pig molecular breeding.

## Materials and methods

### Ethics statement

The guidelines of the experimental animal management of China Agricultural University (CAU) were followed throughout the study, and the experimental protocols were approved by the Experimental Animal Care and Use Committee of CAU.

### Animal and sample collection

A total of 243 six-month-old sows (Landrace × Large White breed background), nearly 100 kg (± 5 kg) weight, were collected from Jining Anxin Breeding Co., Ltd in Shandong for evaluating BFT and collecting ear tissue samples. Pigs collected in this study were unrelated and in good health. All pigs were raised under consistent conditions, including farm, feeding, and management, etc. Ear tissue samples were collected and stored in 1.5 mL centrifuge tubes with 75% ethanol at −20 °C.

### Backfat thickness measurement

Pig BFT was measured with a live backfat instrument (RENCO, USA) according to the user’s guide. Briefly, each pig was fixed in a crate, and the measurement position was selected at a point 50 mm from the midline of the back between the penultimate third and fourth ribs. Before the detection, the pig hair at the measured position was cut off and applied an appropriate amount of coupling agent. Then, the pig BFT information was detected by attaching the probe coated with the coupling agent vertically and tightly to the measurement position.

### Genome extraction and mtDNA sequencing

Genomic DNA was extracted using Tissue/Cell Genome DNA Extraction Kits (Aidlab, DN08) according to the manufacturer’s instruction. The primer pair, forward primer 5′-CAAGACTCAAGGAAGGAGACT-3′ from nucleotide (nt) 16543 to nt 16563 and reverse primer 5′-AACCTGTGTGTTTATGGAGC-3′ from nt 1234 to nt 1253, was used to amplify the mtDNA D-loop region, which was designed in our previous report.^33^ The polymerase chain reaction (PCR) amplification was performed in a 30 µL reaction buffer containing 15 µL of 2 × A9 LongHiFi PCR MasterMix (Aidlab, PC84), 1 µL of each forward and reverse primers (10 µM), 1 µL extracted DNA and 12 µL double-distilled H_2_O. The PCR program was pre-denatured at 95 °C for 3 min, followed by 30 circles of 95 °C for 10 s, 56 °C for 15 s and 72 °C for 20 s with a final 72 °C for 2 min. After electrophoresis, PCR products were sequenced through Sanger sequencing method by Beijing Qingke Technology Co., Ltd. The complete porcine mitochondrial genome sequence (GenBank Accession no. NC_000845.1) was used as the reference sequence for sequence alignment. The mtDNA polymorphisms in the D-loop region were identified through sequence alignments of 243 samples using the software MEGA7 (version 7.0.26).

### Statistical analysis

Mitochondrial genome haplotypes were classified and sorted by DnaSP (version 5.10.01) and FaBox online software (http://users-birc.au.dk/biopv/php/fabox/). The Median-joining method in the Network 5 software was used to perform cluster analysis on haplotypes, and haplotype groups were classified according to mutated positions among haplotypes. The association between mtDNA polymorphism/haplotype/haplogroup and pig BFT was analysed by the analysis of variance (ANOVA), followed by Duncan’s multiple-range test using the general linear model procedure of software SAS (version 8.2). Results were given as the mean ± standard error of the mean (SEM) and two-sided *P* <  0.05 or 0.01 was considered significant or extremely significant, respectively.

## Result

### Morphological observations on backfat thickness

**Figure 1.**
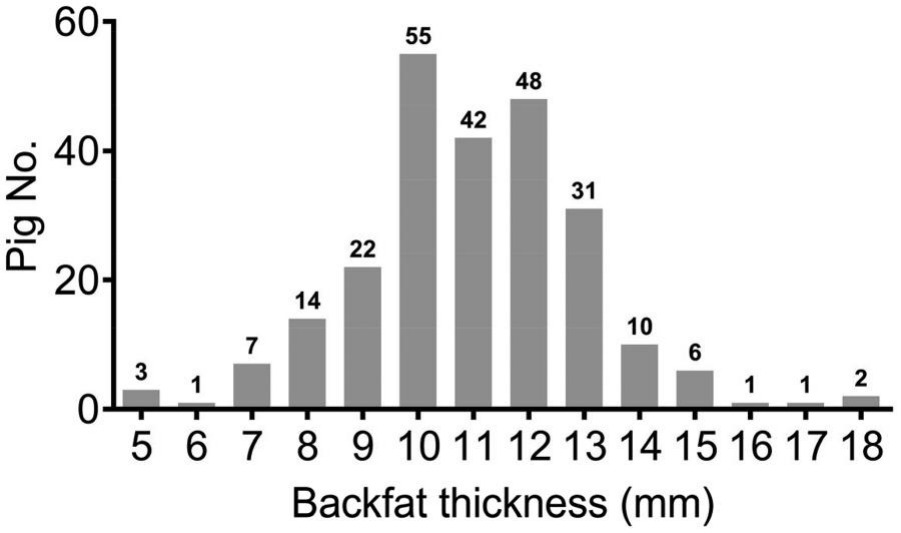
The distribution plot of the backfat thickness of 243 pigs.

As shown in Figure 1, the average level of BFT per pig was approximately 11 mm, whereas the value of BFT varied considerably, ranging from 5 mm to 18 mm per pig. Among these pigs, 55 of which exhibited 10 mm BFT, followed by 48 pigs owned 12 mm BFT and 42 pigs contained 11 mm BFT, which showed that pigs with 11 to 12 mm BFT accounted for 60% of the total (Figure 1).

### Association analysis of mtDNA polymorphisms and backfat thickness

To investigate the great differences in BFT among pigs, we detected mutations in the mtDNA D-loop region of pigs and analysed the potential effect on BFT through association analysis. A total of 48 polymorphic sites were found in the mtDNA D-loop region among 243 sows (Table S1), 30 of which were associated with BFT (Table 1). Among them, the m.794A > G mutation had the largest difference value, with differences of more than 2.5 mm in the BFT between A and G carriers (*P* <  0.001).

**Table 1.** Mitochondrial DNA polymorphic sites associated with porcine backfat thickness.

Site	Nucleotide (Number, BFT)	Nucleotide (Number, BFT)	D-value (mm)	*P* value
109	C (*n* = 148, 10.73 ± 0.17)	T (*n* = 95, 11.27 ± 0.20)	0.54	0.04354
131	A (*n* = 149, 10.72 ± 0.17)	G (*n* = 94, 11.29 ± 0.20)	0.57	0.03721
137	- (*n* = 149, 10.72 ± 0.17)	C (*n* = 94, 11.29 ± 0.20)	0.57	0.03721
145	C (*n* = 94, 11.29 ± 0.20)	T (*n* = 149, 10.72 ± 0.17)	0.57	0.03721
153	C (*n* = 94, 11.29 ± 0.20)	T (*n* = 149, 10.72 ± 0.17)	0.57	0.03721
158	A (*n* = 94, 11.29 ± 0.20)	G (*n* = 149, 10.72 ± 0.17)	0.57	0.03721
181	C (*n* = 98, 10.62 ± 0.23)	T (*n* = 145, 11.16 ± 0.16)	0.54	0.04550
241	C (*n* = 131, 10.63 ± 0.19)	T (*n* = 112, 11.31 ± 0.18)	0.68	0.00906
294	A (*n* = 94, 11.29 ± 0.20)	G (*n* = 149, 10.72 ± 0.17)	0.57	0.03721
306	C (*n* = 94, 11.29 ± 0.20)	T (*n* = 149, 10.72 ± 0.17)	0.57	0.03721
390	C (*n* = 94, 11.29 ± 0.20)	T (*n* = 149, 10.72 ± 0.17)	0.57	0.03721
452	C (*n* = 176, 11.14 ± 0.15)	T (*n* = 67, 10.42 ± 0.24)	0.72	0.01367
704	A (*n* = 94, 11.29 ± 0.20)	G (*n* = 149, 10.72 ± 0.17)	0.57	0.03721
706	A (*n* = 94, 11.29 ± 0.20)	G (*n* = 149, 10.72 ± 0.17)	0.57	0.03721
714	A (*n* = 16, 8.81 ± 0.48)	G (*n* = 227, 11.09 ± 0.13)	2.28	0.00001
722	A (*n* = 16, 8.81 ± 0.48)	G (*n* = 227, 11.09 ± 0.13)	2.28	0.00001
734	A (*n* = 16, 8.81 ± 0.48)	G (*n* = 227, 11.09 ± 0.13)	2.28	0.00001
742	A (*n* = 16, 8.81 ± 0.48)	G (*n* = 227, 11.09 ± 0.13)	2.28	0.00001
752	A (*n* = 16, 8.81 ± 0.48)	G (*n* = 227, 11.09 ± 0.13)	2.28	0.00001
762	A (*n* = 16, 8.81 ± 0.48)	G (*n* = 227, 11.09 ± 0.13)	2.28	0.00001
772	A (*n* = 16, 8.81 ± 0.48)	G (*n* = 227, 11.09 ± 0.13)	2.28	0.00001
782	A (*n* = 16, 8.81 ± 0.48)	G (*n* = 227, 11.09 ± 0.13)	2.28	0.00001
794	A (*n* = 12, 8.50 ± 0.58)	G (*n* = 231, 11.07 ± 0.13)	2.57	0.00002
802	A (*n* = 14, 8.79 ± 0.54)	G (*n* = 229, 11.07 ± 0.13)	2.28	0.00004
812	A (*n* = 14, 8.79 ± 0.54)	G (*n* = 229, 11.07 ± 0.13)	2.28	0.00004
822	A (*n* = 15, 8.87 ± 0.51)	G (*n* = 228, 11.08 ± 0.13)	2.21	0.00004
834	A (*n* = 11, 8.64 ± 0.62)	G (*n* = 232, 11.05 ± 0.13)	2.41	0.00011
1089	C (*n* = 149, 10.72 ± 0.17)	T (*n* = 94, 11.29 ± 0.20)	0.57	0.03721
1096	A (*n* = 94, 11.29 ± 0.20)	G (*n* = 149, 10.72 ± 0.17)	0.57	0.03721
1146	C (*n* = 158, 10.73 ± 0.16)	T (*n* = 85, 11.34 ± 0.22)	0.61	0.02599

Data of BFT were shown as mean (mm) ± SEM.

### Association analysis of mtDNA haplotype/haplogroup and backfat thickness

**Figure 2.**
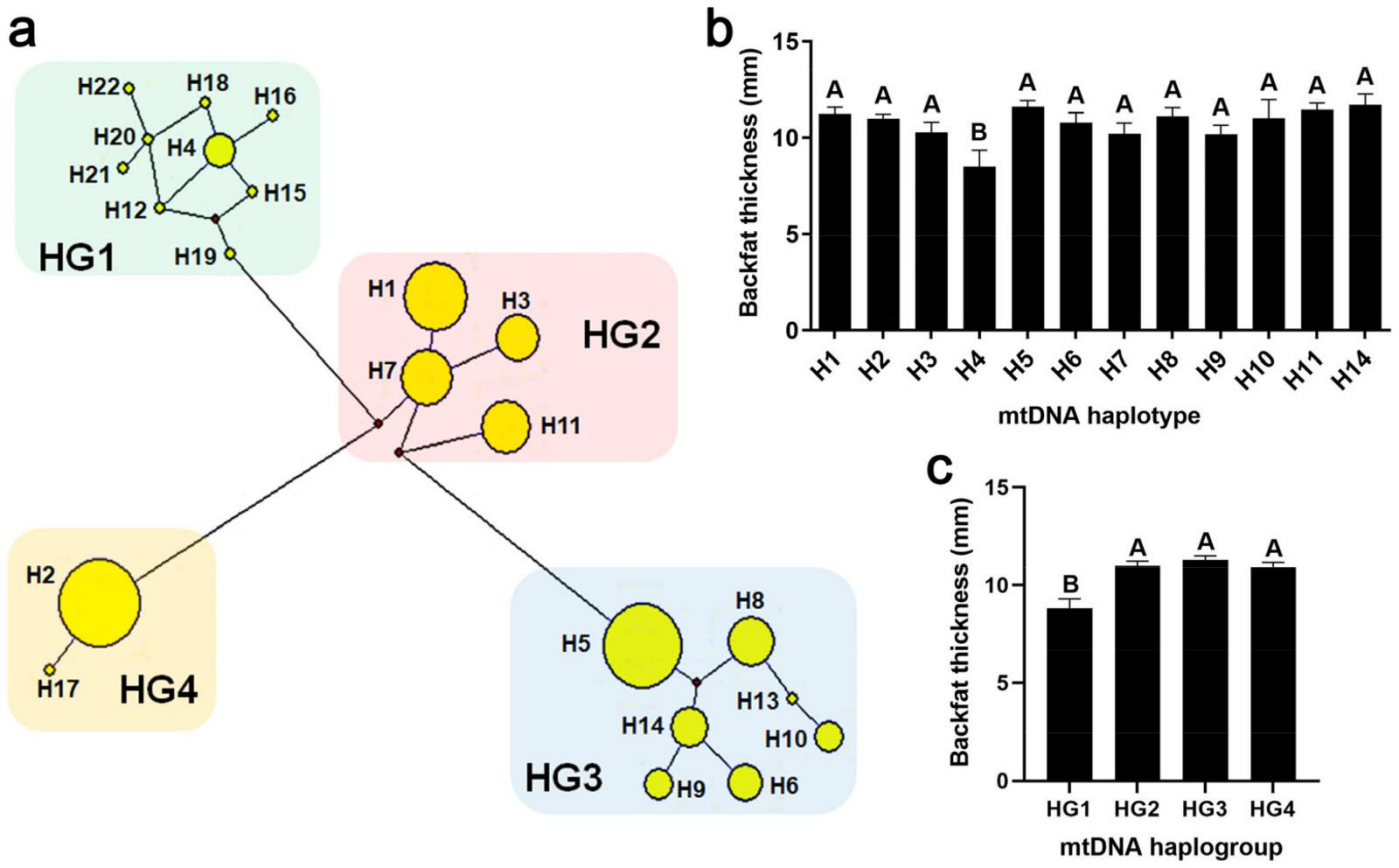
Cluster analysis of mtDNA haplotypes and their correlation with backfat thickness. (a) Cluster analysis of mtDNA haplotypes (H): the 22 haplotypes were clustered into four haplogroups (HG), HG1-HG4. The size of yellow circles indicated the number of pigs belonged to each haplotype. (b) Association analysis of haplotype and backfat thickness. Only haplotypes that were found in more than three individuals were analysed. (c) Association analysis of haplogroup and backfat thickness. Data were shown as mean ± SEM. Different capital letters above columns indicated extremely significant differences, *P* < 0.01.

Based on 48 polymorphic sites in mtDNA D-loop region, 22 haplotypes were identified, which were named H1-H22 (Figure 2(a) and Table S1). The sequence information of every haplotype (H1-H22) was uploaded to the GenBank of NCBI, and accession numbers were as follows: MT407548-MT407569. Association analysis presented that the BFT in pigs with H4 was significantly lower than that in pigs with other haplotypes (*P* < 0.01), and specimens H4 (∼8.5 mm) and H14 (∼11.7 mm) contained the minimum and maximum BFT of pigs, respectively, which existed a 3.20 mm difference on BFT between them (Figure 2(b) and Table S2).

The 22 haplotypes were grouped into four diverse haplogroups (named HG1-HG4, Figure 2(a)) according to mutated positions among haplotypes (Table S1). Notably, haplotypes H4, H12, H15, H16, H18, H19, H20, H21 and H22 were clustered into HG1, whereas haplotypes H1, H3, H9, H7 and H11 were in HG2, haplotypes H5, H6, H8, H9, H10, H13 and H14 were in HG3, haplotypes H2 and H17 were in HG4 (Figure 2(a)). As shown in Figure 2c and Table S2, pigs with HG2, HG3 or HG4 exhibited higher BFT than pigs with HG1 (*P* < 0.01), and the difference of BFT between HG1 and HG3 was the highest, reaching nearly 2.48 mm (*P* < 0.01).

By examining the frequencies of the thirty mtDNA polymorphic sites correlated with BFT, the imbalanced distribution among the four haplogroups were revealed (Table 2). The frequencies of thirteen mtDNA mutations (m.714A > G, m.722A > G, m.734A > G, m.742A > G, m.752A > G, m.762A > G, m.772A > G, m.782A > G, m.794A > G, m.802A > G, m.812A > G, m.822A > G and m.834A > G) with lower BFT (Table 1) were all very high in HG1, and ranged from 0.69 to 1.00, whereas the reverse results were found in other three haplogroups (HG2-HG4) (Table 2). Among the thirteen mutations, eight of which (m.714A > G, m.722A > G, m.734A > G, m.742A > G, m.752A > G, m.762A > G, m.772A > G and m.782A > G) were completely linked and formed the basic distinction between HG1 and other three haplogroups (HG2-HG4).

**Table 2.** Frequency of mitochondrial DNA polymorphic sites in haplogroups.

Mutation	Nucleotide	Frequency			
		HG1 (*n* = 16)	HG2 (*n* = 82)	HG3 (*n* = 94)	HG4 (*n* = 51)
m.109C > T	C	0.94	1.00	0.00	1.00
m.131A > G	A	1.00	1.00	0.00	1.00
m.137->C	–	1.00	1.00	0.00	1.00
m.145C > T	C	0.00	0.00	1.00	0.00
m.153C > T	C	0.00	0.00	1.00	0.00
m.158A > G	A	0.00	0.00	1.00	0.00
m.181C > T	C	1.00	1.00	0.00	0.00
m.241C > T	C	1.00	0.78	0.00	1.00
m.294A > G	A	0.00	0.00	1.00	0.00
m.306C > T	C	0.00	0.00	1.00	0.00
m.390C > T	C	0.00	0.00	1.00	0.00
m.452C > T	C	0.00	1.00	1.00	0.00
m.704A > G	A	0.00	0.00	1.00	0.00
m.706A > G	A	0.00	0.00	1.00	0.00
m.714A > G	A	1.00	0.00	0.00	0.00
m.722A > G	A	1.00	0.00	0.00	0.00
m.734A > G	A	1.00	0.00	0.00	0.00
m.742A > G	A	1.00	0.00	0.00	0.00
m.752A > G	A	1.00	0.00	0.00	0.00
m.762A > G	A	1.00	0.00	0.00	0.00
m.772A > G	A	1.00	0.00	0.00	0.00
m.782A > G	A	1.00	0.00	0.00	0.00
m.794A > G	A	0.75	0.00	0.00	0.00
m.802A > G	A	0.88	0.00	0.00	0.00
m.812A > G	A	0.88	0.00	0.00	0.00
m.822A > G	A	0.94	0.00	0.00	0.00
m.834A > G	A	0.69	0.00	0.00	0.00
m.1089C > T	C	1.00	1.00	0.00	1.00
m.1096A > G	A	0.00	0.00	1.00	0.00
m.1146C > T	C	1.00	1.00	0.10	1.00

## Discussion

The backfat serves as a reliable indicator of fat deposition ability, animal condition and overall health.^34–36^ It is closely associated with both productive and reproductive traits, such as lean percentage,^37^^,^^38^ inflammation and litter size,^39–42^ and regarded as a significant parameter in pig breeding programmes. BFT is an important quantitative trait that varies among pig breeds. As a carcass trait, it has high heritability and is mainly regulated by multiple factors, mainly including genetics, environment, and nutrition.^31^^,^^43–45^ One common approach for BFT selection process is to conduct regular measurements of BFT using ultrasound or other precise measuring tools.^46^ This accurate assessment of BFT at different growth stages provides a rough evaluation of an individual’s genetic potential. As genetics progresses, newer methods like marker-assisted selection (MAS) and genomic selection (GS) have been introduced for selection and breeding.^47–49^ These methods enable the identification of pigs with superior genetic potential for fat deposition, facilitating more targeted and efficient breeding decisions. However, it is important to note that these projects primarily focus on analyzing nuclear genome and overlook the significant contributions made by mtDNA.

Mitochondria are closely related to metabolism, energy generation and cellular homeostasis, affecting animal developments, such as skeletal muscle growth, fat metabolism and deposition, maturation of germ cells, and even disease resistance.^13^^,^^50^^,^^51^ As the important extrachromosomal genetic material, mtDNA exists in mitochondria and codes peptides that involved in respiratory chain complexes. MtDNA polymorphisms are one of the main factors affecting mitochondrial function through affecting mtDNA replication and expression, and the function of mitochondrial complexes.^13^^,^^52^^,^^53^ In the mitochondrial genome, the D-loop region is the core domain for regulating mtDNA replication, transcription and stability through combining various effectors.^54^ Mutations in mtDNA D-loop may disturb these biological processes and lead to mitochondrial dysfunction.^53^ Therefore, the correlation between polymorphisms of the mtDNA D-loop and pig BFT was analyzed in the present study, which would like to fill the gap in extranuclear genetic effect on BFT. The results found 48 mutations in 243 pig samples, including eight sites (m.714A > G, m.722A > G, m.734A > G, m.742A > G, m.752A > G, m.762A > G, m.772A > G and m.782A > G) that were completely linked and formed the basic differentiation of the haplogroup HG1 with another three haplogroups (HG2, HG3 and HG4) (Figure 2 and Table 2). The H4, belonged to HG1, exhibited the lowest BFT among haplotypes (Figure 2). And the point mutation m.794A > G showed the largest BFT difference: the BFT in m.794A carriers was over 2.5 mm less than that in m.794G sows (Table 1). As we can see in Table 2, the frequency of A at the m.794 site was very high in HG1, which reached 0.75; however, no A mutation was detected in the other three haplogroups. These data suggested that the mutation m.794A > G or haplotype H4 probably acted prominently in backfat deposition. The results of the present study revealed that mtDNA polymorphic sites, haplotypes and haplogroups were associated with live BFT of pigs, allowing us to suggest that mtDNA molecular could be used as candidate gene markers for animal breeding, reflecting BFT. Our previous study has identified that thirteen mtDNA polymorphic sites are significantly associated with the number of oocytes in pigs with Landrace × Large White breed background.^33^ Among these mutations, 11 of which (m.109C > T, m.131A > G, m.145C > T, m.153C > T, m.158A > G, m.241C > T, m.294A > G, m.306C > T, m.390C > T, m.704A > G and m.706A > G) were identified their associations with BFT in this study (Table 1). Notably, these mutations exhibited opposing effects between BFT and oocyte number, indicating that mtDNA mutations associated with higher oocyte number were correlated with lower BFT. This observation suggested that reducing BFT within a certain range could be beneficial for improving the reproductive performance of pigs, which was consistent with previous studies.^40^^,^^42^

The function of mitochondria is regulated by the collaboration of proteins encoded by nuclear and mitochondrial genes. In this study, the Landrace × Large White breed pigs were selected for analysis, which provided a relative high similarity in nuclear genome among pigs; but they cannot be completely identical, which might have slight interferences to identify mtDNA effects on BFT. Therefore, in the next study, we will construct cybrids cells, mtDNA point mutation cells and transmitochondrial pigs to provide more rigorous evidence for revealing the effect of mtDNA mutations/haplotypes on pig traits.

## Conclusion

Specific mtDNA mutations, haplotypes and haplogroups were characterized and linked with the porcine BFT trait. As the BFT is a quantitative trait with high heritability, mtDNA polymorphisms could be useful in marker-assisted selection for greater predictability of carcass traits. For further study, these mtDNA polymorphisms or haplotypes should be put into post-association validation and might be used as genetic markers in porcine selection and breeding projects.

## Supplementary Material

Supplemental Material

Supplemental Material

## Data Availability

Sequences of mtDNA haplotypes were submitted and deposited in the GenBank database.

## Supplementary material

Table S1. MtDNA mutations among twenty-two haplotypes.

Table S2. Backfat thickness values for each mtDNA haplotype/haplogroup.
