# Supplementary material for: Association analysis of mitochondrial genome polymorphisms with backfat thickness in pigs
Source: Anim Biotechnol. 2023 Nov 15;35(1):2272172. doi: 10.1080/10495398.2023.2272172 (PMC12674358; doi:10.1080/10495398.2023.2272172)
Supplement: Supplemental Material [file LABT_A_2272172_SM9248.docx]

Table S2. Backfat thickness values for each mtDNA haplotype/haplogroup.

| No. | Mean | SEM |
| --- | --- | --- |
| Haplotype |  |  |
| H1 | 11.24 | 0.34 |
| H2 | 10.98 | 0.24 |
| H3 | 10.27 | 0.54 |
| H4 | 8.50 | 0.85 |
| H5 | 11.60 | 0.32 |
| H6 | 10.78 | 0.52 |
| H7 | 10.20 | 0.57 |
| H8 | 11.12 | 0.43 |
| H9 | 10.17 | 0.48 |
| H10 | 11.00 | 0.97 |
| H11 | 11.44 | 0.35 |
| H14 | 11.70 | 0.56 |
| Haplogroup |  |  |
| HG1 | 8.81 | 0.48 |
| HG2 | 10.98 | 0.24 |
| HG3 | 11.29 | 0.20 |
| HG4 | 10.92 | 0.24 |
