# Supplementary material for: Association analysis of mitochondrial genome polymorphisms with backfat thickness in pigs
Source: Anim Biotechnol. 2023 Nov 15;35(1):2272172. doi: 10.1080/10495398.2023.2272172 (PMC12674358; doi:10.1080/10495398.2023.2272172)
Supplement: Supplemental Material [file LABT_A_2272172_SM9179.docx]

Table S1. MtDNA mutations among twenty-two haplotypes.

| No. | Site | mtDNA haplotype | | | | | | | | | | | | | | | | | | | | | |
| --- | --- | --- | --- | --- | --- | --- | --- | --- | --- | --- | --- | --- | --- | --- | --- | --- | --- | --- | --- | --- | --- | --- | --- |
|  |  | H 1 | H 2 | H 3 | H 4 | H 5 | H 6 | H 7 | H 8 | H 9 | H 10 | H 11 | H 12 | H 13 | H 14 | H 15 | H 16 | H 17 | H 18 | H 19 | H 20 | H 21 | H 22 |
| 1 | 36 | A | A | A | A | A | A | A | A | A | A | A | A | A | A | A | A | T | A | A | A | A | A |
| 2 | 109 | C | C | C | C | T | T | C | T | T | T | C | C | T | T | C | C | C | C | C | C | C | T |
| 3 | 124 | A | A | A | A | A | T | A | T | T | T | A | A | T | T | A | A | A | A | A | A | A | A |
| 4 | 131 | A | A | A | A | G | G | A | G | G | G | A | A | G | G | A | A | A | A | A | A | A | A |
| 5 | 137 | - | - | - | - | C | C | - | C | C | C | - | - | C | C | - | - | - | - | - | - | - | - |
| 6 | 143 | A | T | A | A | A | A | A | A | A | A | A | A | A | A | A | A | T | A | A | A | A | A |
| 7 | 145 | T | T | T | T | C | C | T | C | C | C | T | T | C | C | T | T | T | T | T | T | T | T |
| 8 | 153 | T | T | T | T | C | C | T | C | C | C | T | T | C | C | T | T | T | T | T | T | T | T |
| 9 | 158 | G | G | G | G | A | A | G | A | A | A | G | G | A | A | G | G | G | G | G | G | G | G |
| 10 | 181 | C | T | C | C | T | T | C | T | T | T | C | C | T | T | C | C | T | C | C | C | C | C |
| 11 | 241 | C | C | C | C | T | T | C | T | T | T | T | C | T | T | C | C | C | C | C | C | C | C |
| 12 | 279 | T | C | T | C | C | C | T | C | C | C | T | C | C | C | C | C | C | C | C | C | C | C |
| 13 | 288 | A | A | A | A | A | G | A | A | A | A | A | A | A | A | A | A | A | A | A | A | A | A |
| 14 | 294 | G | G | G | G | A | A | G | A | A | A | G | G | A | A | G | G | G | G | G | G | G | G |
| 15 | 306 | T | T | T | T | C | C | T | C | C | C | T | T | C | C | T | T | T | T | T | T | T | T |
| 16 | 323 | T | T | T | T | T | C | T | C | C | C | T | T | C | C | T | T | T | T | T | T | T | T |
| 17 | 387 | A | A | G | A | A | A | A | A | A | A | A | A | A | A | A | A | A | A | A | A | A | A |
| 18 | 390 | T | T | T | T | C | C | T | C | C | C | T | T | C | C | T | T | T | T | T | T | T | T |
| 19 | 405 | T | C | T | T | T | T | T | T | C | T | T | T | T | T | T | T | C | T | T | T | T | T |
| 20 | 452 | C | T | C | T | C | C | C | C | C | C | C | T | C | C | T | T | T | T | T | T | T | T |
| 21 | 501 | A | A | A | A | A | A | A | G | A | G | G | A | G | A | A | A | A | A | A | A | A | A |
| 22 | 575 | G | G | G | G | G | A | G | G | A | G | G | G | G | A | G | G | G | G | G | G | G | G |
| 23 | 652 | C | C | C | C | C | C | C | C | C | C | C | C | C | C | C | - | C | C | C | C | C | C |
| 24 | 692 | A | G | G | G | G | G | G | G | G | G | G | G | G | G | G | G | G | G | G | G | G | G |
| 25 | 704 | G | G | G | G | A | A | G | A | A | A | G | G | A | A | G | G | G | G | G | G | G | G |
| 26 | 706 | G | G | G | G | A | A | G | A | A | A | G | G | A | A | G | G | G | G | G | G | G | G |
| 27 | 714 | G | G | G | A | G | G | G | G | G | G | G | A | G | G | A | A | G | A | A | A | A | A |
| 28 | 722 | G | G | G | A | G | G | G | G | G | G | G | A | G | G | A | A | G | A | A | A | A | A |
| 29 | 724 | G | G | G | G | G | G | G | G | G | A | G | G | G | G | G | G | G | G | G | G | G | G |
| 30 | 726 | G | G | G | G | G | G | G | G | G | A | G | G | A | G | G | G | G | G | G | G | G | G |
| 31 | 734 | G | G | G | A | G | G | G | G | G | G | G | A | G | G | A | A | G | A | A | A | A | A |
| 32 | 742 | G | G | G | A | G | G | G | G | G | G | G | A | G | G | A | A | G | A | A | A | A | A |
| 33 | 752 | G | G | G | A | G | G | G | G | G | G | G | A | G | G | A | A | G | A | A | A | A | A |
| 34 | 762 | G | G | G | A | G | G | G | G | G | G | G | A | G | G | A | A | G | A | A | A | A | A |
| 35 | 772 | G | G | G | A | G | G | G | G | G | G | G | A | G | G | A | A | G | A | A | A | A | A |
| 36 | 782 | G | G | G | A | G | G | G | G | G | G | G | A | G | G | A | A | G | A | A | A | A | A |
| 37 | 792 | G | G | G | G | G | G | G | G | G | G | G | G | G | G | G | G | G | G | G | G | A | G |
| 38 | 794 | G | G | G | A | G | G | G | G | G | G | G | A | G | G | A | A | G | G | A | G | G | G |
| 39 | 802 | G | G | G | A | G | G | G | G | G | G | G | A | G | G | A | A | G | A | G | A | G | A |
| 40 | 812 | G | G | G | A | G | G | G | G | G | G | G | A | G | G | G | A | G | A | G | A | A | A |
| 41 | 822 | G | G | G | A | G | G | G | G | G | G | G | A | G | G | A | A | G | A | G | A | A | A |
| 42 | 832 | G | G | G | G | G | G | G | G | G | G | G | G | G | G | G | G | G | G | G | G | A | G |
| 43 | 834 | G | G | G | A | G | G | G | G | G | G | G | G | G | G | A | A | G | A | G | G | G | G |
| 44 | 992 | T | T | T | T | T | T | T | T | C | T | T | T | T | T | T | T | T | T | T | T | T | T |
| 45 | 1029 | D | I | D | D | D | D | D | D | D | D | D | D | D | D | D | D | I | D | D | D | D | D |
| 46 | 1089 | C | C | C | C | T | T | C | T | T | T | C | C | T | T | C | C | C | C | C | C | C | C |
| 47 | 1096 | G | G | G | G | A | A | G | A | A | A | G | G | A | A | G | G | G | G | G | G | G | G |
| 48 | 1146 | C | C | C | C | T | C | C | T | T | T | C | C | T | T | C | C | C | C | C | C | C | C |

The reference mtDNA sequence is NC000854.1 from NCBI. The “I” or “D” mutation at 1029 site meant "TAAAACATTA" insertion or deletion, respectively.
